# Whole-exome sequencing identifies de novo mutation in the *COL1A1* gene to underlie the severe osteogenesis imperfecta

**DOI:** 10.1186/s40246-015-0028-0

**Published:** 2015-05-10

**Authors:** Katre Maasalu, Tiit Nikopensius, Sulev Kõks, Margit Nõukas, Mart Kals, Ele Prans, Lidiia Zhytnik, Andres Metspalu, Aare Märtson

**Affiliations:** Clinic of Traumatology and Orthopaedics, University of Tartu, Puusepa 8, 51014 Tartu, Estonia; Clinic of Traumatology and Orthopaedics, Tartu University Hospital, Puusepa 8, 51014 Tartu, Estonia; Estonian Genome Centre, University of Tartu, Riia 23b, Tartu, 51010 Estonia; Institute of Molecular and Cell Biology, University of Tartu, Riia 23b, Tartu, 51010 Estonia; Department of Pathophysiology, University of Tartu, Ravila 19, Tartu, 50411 Estonia; Estonian Biocentre, Riia 23b, 51010 Tartu, Estonia

**Keywords:** Osteogenesis imperfecta, Type I collagen, OI genotype–phenotype, *COL1A1*, *De novo* mutation

## Abstract

**Background:**

Osteogenesis imperfecta (OI) comprises a clinically and genetically heterogeneous group of connective tissue disorders, characterized by low bone mass, increased bone fragility, and blue-gray eye sclera. OI often results from missense mutations in one of the conserved glycine residues present in the Gly-X-Y sequence repeats of the triple helical region of the collagen type I α chain, which is encoded by the *COL1A1* gene. The aim of the present study is to describe the phenotype of OI II patient and a novel mutation, causing current phenotype.

**Results:**

We report an undescribed de novo *COL1A1* mutation in a patient affected by severe OI. After performing the whole-exome sequencing in a case parent–child trio, we identified a novel heterozygous c.2317G > T missense mutation in the *COL1A1* gene, which leads to p.Gly773Cys transversion in the triple helical domain of the collagen type I α chain. The presence of the missense mutation was confirmed with the Sanger sequencing.

**Conclusions:**

Hereby, we report a novel mutation in the *COL1A1* gene causing severe, life threatening OI and indicate the role of de novo mutation in the pathogenesis of rare familial diseases. Our study underlines the importance of exome sequencing in disease gene discovery for families where conventional genetic testing does not give conclusive evidence.

## Introduction

Osteogenesis Imperfecta (OI), or “brittle bone” disease, is a heritable disorder of collagen type I metabolism with a generalized involvement of connective tissues. Collagen is the most abundant protein in mammals, constituting a quarter of the total protein weight [1]. Collagens are grouped into families based on their structural and functional features. Type I collagen is the major protein in bone, skin, tendon, ligament, sclera and cornea tissues, blood vessels, and hollow organs ENREF 2 [2]. OI is mostly caused by quantitative or qualitative collagen type I defects. The condition is characterized by low bone mass, bone fragility, and often short stature. Extraskeletal manifestations may include blue-gray eye sclera and dental abnormalities. The clinical severity varies widely from nearly asymptomatic forms with a mild predisposition to fractures, normal stature, and normal lifespan to profoundly disabling and even lethal [3, 4].

The pathogenetic approach to OI is changed with the recent identification of non-collagenous genes, mutations in which may cause OI. In general, a clear genotype-phenotype correlation does not exist. General rules for genotype-phenotype correlations have been published only in *COL1A1/2*-related OI [5]. Approximately 90 % of individuals affected with OI are heterozygous for a causative variant in one of the two genes, *COL1A1* or *COL1A2*, which encode the pro-1(I) and pro-2(I) chains of type I procollagen, respectively [6].

The proportion of cases caused by a de novo *COL1A1* or *COL1A2* mutation varies according to the severity of the disease. Approximately 60 % of cases of classic non-deforming OI with blue sclerae or common variable OI with normal sclerae, virtually 100 % of perinatally lethal OI, and close to 100 % of progressively deforming OI are caused by de novo mutations [7, 8].

In 1979, a classification of OI was introduced by David Sillence and the disease was divided into four types with a wide spectrum of clinical features, where OI type II is the most severe and prenatally lethal form of the disorder [9]. Initial classification, based on clinical and histological manifestations, was extended into five distinct types of OI [10]. By now, genetic studies described various OI phenotypes, and genetic OI classification is broadened up to 15 different OI types, according to the affected gene. In addition to collagen genes, OI is caused by mutations in the *CRTAP* (OI VII), *LEPRE 1* (OI VIII), *BMP1* (OI XIII), *TMEM38B* (OI XIV), *IFITM5* (OI V), *SERPINH1* (OI X), *WNT1* (OI XV), *SP7* (OI XII), *PPIB* (OI IX), *SERPINF1* (OI VI), *FKBP10* (OI XI) genes [11–14].

In addition to big genotype diversity, the phenotype manifestations may vary not only among representatives of the same OI type but also among carriers of the same mutation and even affected members of the same family. Some of the OI forms represent transitional phenotypes between different forms. Therefore, the genotype-phenotype relationships and clinical manifestations of OI are still difficult to explain.

The aim of present study is to describe the phenotype of OI II patient, with severe life threatening bone fragility, and report a novel mutation, causing current phenotype. We strongly believe that new additional information on current transitional OI form will deepen the understanding of the pathogenesis of the brittle bone disease.

## Materials and methods

**Fig. 1:**
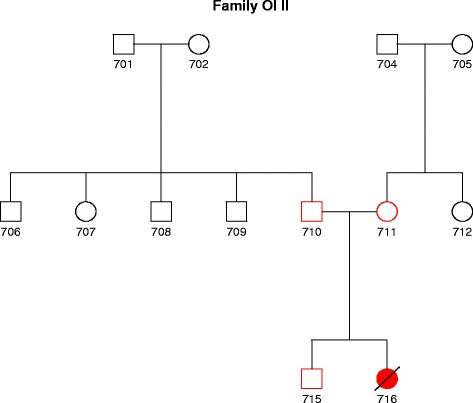
Pedigree structure of an Estonian family affected with type II OI. DNA was collected from father (710), mother (711), brother (715), and proband (716)

The Ethics Review Committee on Human Research of the University of Tartu approved the study and the participants, and their legal representatives gave prior consent to participate in the study and publish the results. Blood samples were obtained from the proband (716), her brother (715), and parents (710 and 711) from a family without previous history of OI (Fig. 1). Genomic DNA was extracted from EDTA-preserved blood according to standard high-salt extraction methods and stored at −80 °C.

The proband (716) was the first child (first pregnancy, first delivery) of non-consanguineous Estonian parents; age of the mother and father were 23 and 27 years, respectively. The parents were healthy without history of chronic or clinically significant diseases.

The girl was born with totally soft skull, the head was large (diameter 43 cm), and bones in occipital region were not palpable (skinhead). Her head was flat, being collapsed from front to back direction. She has exophthalmia and blue sclerae. The newborn had disproportional growth retardation (limbs > body): short, bowed arms and legs, and both hips were hyperflexed and turned outward.

Total skinhead and only partially developed upper part of parietal and mandibular bones were detected in Babygram X-ray investigation on date of birth. All long bones were extremely osteopenic; she had accordion-type ribs, and several fractures in different healing stages were detected in every bone. In addition, fresh right humeral fracture and left tibial fracture were confirmed.

In genetic counseling, at the age of 3 days, the baby was diagnosed with osteogenesis imperfecta type II based on clinical signs and X-ray data. As family history was negative for the OI, de novo autosomal dominant mutation was suspected.

### Exome sequencing

Whole-exome sequencing was performed on an affected child and both unaffected parents at the NGS core facility of the Estonian Genome Center, University of Tartu. Exome capture was performed using the TruSeq Exome Enrichment kit (Illumina) following the manufacturer’s protocol. The captured libraries were sequenced with Illumina HiSeq2000 with 100-bp paired-end reads. Over 10 Gb of sequence was generated from each individual, resulting in a coverage depth of 84× for both parents and 87× for an affected child. Sequence reads were aligned to the human reference genome (hg19, GRCh37) with the Burrows-Wheeler Aligner (BWA, version 0.6.1) [15]. Single-nucleotide substitutions and small indel variants were called with SAM tools (version 0.1.18), Picard tools (version 1.60), and a Genome Analysis Toolkit (GATK, version 1.5.21) [16, 17]. Genotypes were called at all positions with high-quality sequence bases and filtered to retain SNPs and insertion-deletions with Phred-like quality scores of at least 20. We focused on non-synonymous and canonical splice-site variants being absent from public datasets (including dbSNP135 and the 1000 Genomes Project) and in-house exome and full-genome data. We used the PolyPhen-2, SIFT, and Condel software tools to predict the functional effects of mutations [18–20]. Mutation analysis was performed with Sanger sequencing on an affected child (716), on both parents (710, 711), and an unaffected brother (715).

## Results

**Fig. 2:**
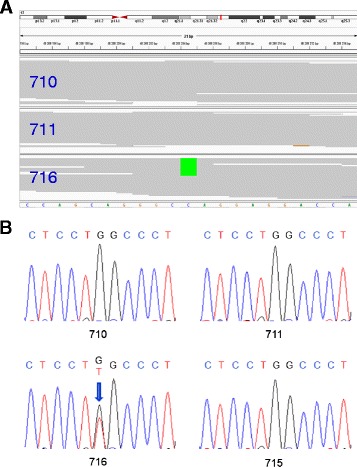
Results of the validation of novel *COL1A1* mutation. **a** The Integrated Genomics Viewer image corresponding to *COL1A1* exon 33–34 de novo variant c.2317G > T (GGC > TGC on „ + “strand). Genomic coordinates are given according to GRCh37/hg19 reference sequence. **b** Validation of the c.2317G > T by Sanger sequencing. Electropherograms of the index patient (716), her mother (711), father (710). Over 10 Gb of sequence was generated from each individual, resulting in a coverage depth of 84× for both parents and 87× for an affected child, and an unaffected brother (715) is shown. C (cytosine) is blue, T (thymine) is red, G (guanine) is black. The position of the heterozygous c.2317G > T mutation is marked by an arrow. The mutation is absent in both parents, confirming its de novo occurrence in the proband

We studied an Estonian family (Fig. 1) of a patient with severe OI in order to identify causative mutation for the disease. Using exome sequencing, we found a novel heterozygous G to T transversion in exon 33/34 at the position 2317 of the *COL1A1* gene. This mutation leads to the substitution of glycine to cysteine at residue 773 in the triple helical domain of the alpha-1 chain of type I collagen. In the other previously known 15 AR OI genes, no potential pathogenic variants were found. The identified mutation was absent in both parents, demonstrating that this variant arose as de novo in the proband. Validation by Sanger sequencing confirmed that the c.2317G > T (p.Gly773Cys) mutation was present in a heterozygous state in the index patient only (Fig. 2). This missense mutation affects a highly conserved amino acid (phyloP score 5.418), and in silico analysis predicted this variant to be deleterious. This mutation was not present neither in the Exome Variant Server of the NHLBI-ESP database or in the 1000 Genomes database. It was also not detected in 221 Estonian control exomes and in full genomes from 87 Estonians.

## Discussion

Here, we report a heterozygous p.Gly773Cys mutation in *COL1A1*, affecting a highly conserved residue in the triple helical domain of collagen type I α chain, as a novel causative variant for severe OI with the lethal outcome. Therefore, given the large number of different genes responsible for OI forms, the genotype-phenotype relationships and clinical manifestations are difficult to explain and present report gives additional information of pathophysiology of OI.

The severity of clinical signs (blue sclerae, totally soft, large and collapsed head; disproportional growth retardation, short and bowed arms and legs, severe breathing failure) and clinical findings (extremely osteopenic bones, several fractures in different healing stages in all bones, deformities, bone development retention) of our patient resembles those of the patients described with the lethal type of OI.

**Fig. 3:**
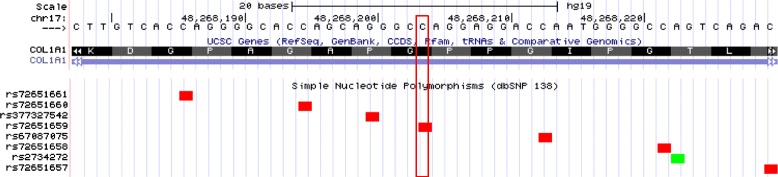
The alignment of DNA and protein sequences of *COL1A1* gene is illustrated. The G to T transversion (red rectangle) in exon 33/34 at the position 2317 of the *COL1A1* gene leads to Gly773 Cys substitution. At the same position, already known SNP (rs72651659) is located, but the known SNP is the G to A transition that leads to Gly773 Ser substitution

Our study found a novel heterozygous G to T transversion in exon at the position 2317 of the *COL1A1* gene (Fig. 3). There is another mutation described in dbSNP database—rs72651659—at the exact same position, with G to A substitution that leads to the substitution of glycine to serine at residue 773. Currently, altogether 756 unique DNA variants (substitutions, insertions, deletions, insertions/deletions, and duplications) in OI patients have been identified in the *COL1A1* gene, and the total number of reported variants is 1352 [11]. The overall pattern of severity of OI phenotypes that result from glycine substitutions in the triple helical domain of the a1(I) chain of type I procollagen is not uniform along the chain. Mutation can be non-lethal, when its position is smaller or equal to 688. If substitution occurs C-terminally to 688, its effect is lethal [21]. Approximately one-third (35.6 %) of all independent glycine substitutions in *COL1A1* are reported to result in lethal type of OI. However, whereas glycine substitutions to valine and to the charged amino acids (aspartic acid, glutamic acid, and arginine) are predominantly lethal (reaching up to 73 % of occurrences for valine), substitutions by the polar residues serine and cysteine have interspersed lethal and non-lethal outcomes with a frequency of lethal cases in ~30 % of occurrences for cysteine [8].

The p.Gly773Cys is located within a long stretch of helical residues 691–823, in which essentially mostly lethal substitutions are identified and also corresponds to Bodian’s “COL1A1 decision tree” about the lethality of mutation [8, 21, 22]. This region correlates with the major ligand binding region 2 (MLBR 2) that extends from helical positions 682–830 in the type I collagen fibril and includes sites that are crucial for collagen self-assembly [8]. This region is important also for interactions of collagen monomers or fibrils with α1β1/α2β1 integrins, matrix metalloproteinases (MMPs), fibronectin, and cartilage oligomeric matrix protein [23]. The Gly773 residue lies within overlapping MMP interaction domain and cell interaction domain with human type I collagen fibril and within ligand binding sites for secreted protein, acidic, and rich in cysteine (SPARC), fibronectin, and MMP1, 12, and 13 [24]. Therefore, mutations in this region interfere severely with the function of protein.

Substitutions of the same glycine residue in *COL1A1* often have independent lethal and non-lethal outcomes. The described p.Gly773Cys mutation was lethal, excluding possibility that this family manifests OI type III. Moreover, neighboring Gly to Cys substitutions are reported to have diverse outcomes. P.Gly770Cys was reported to cause OI type II with a lethal outcome, whereas p.Gly788Cys represents a non-lethal variant causing OI type III/IV [8].

## Conclusions

The present study describes a de novo p.Gly773Cys mutation in *COL1A1* related to the severe OI that gives additional information of pathophysiology of OI. This shows that phenotyping together with genotyping is important to identify special patients and relevant for counseling of parents.

Our study underlines the importance of exome sequencing in disease gene discovery for families where conventional genetic testing does not give conclusive evidence.
